# Modified gateway system for double shRNA expression and Cre/lox based gene expression

**DOI:** 10.1186/1472-6750-11-24

**Published:** 2011-03-22

**Authors:** Nikolina Radulovich, Lisa Leung, Ming-Sound Tsao

**Affiliations:** 1 University Health Network, Ontario Cancer Institute/Princess Margaret Hospital Site, 610 University Ave., Toronto, Ontario, M5G 2M9, Canada; 2 Department of Laboratory Medicine and Pathobiology, Faculty of Medicine, University of Toronto, Medical Sciences Buildings, 1 King's College Circle, Toronto, Ontario, M5 S 1A8, Canada; 3 Department of Medical Biophysics, University Health Network, Ontario Cancer Institute/Princess Margaret Hospital Site, 610 University Ave., Toronto, Ontario, M5G 2M9, Canada

## Abstract

**Background:**

The growing need for functional studies of genes has set the stage for the development of versatile tools for genetic manipulations.

**Results:**

Aiming to provide tools for high throughput analysis of gene functions, we have developed a modified short hairpin RNA (shRNA) and gene expression system based on Gateway Technology. The system contains a series of entry and destination vectors that enables easy transfer of shRNA or cDNA into lentiviral expression systems with a variety of selection or marker genes (i.e. puromycin, hygromycin, green fluorescent protein-EGFP, yellow fluorescent protein-YFP and red fluorescent protein-dsRed2). Our shRNA entry vector pENTR.hU6.hH1 containing two tandem human shRNA expression promoters, H1 and U6, was capable of co-expressing two shRNA sequences simultaneously. The entry vector for gene overexpression, pENTR.CMV.ON was constructed to contain CMV promoter with a multiple cloning site flanked by loxP sites allowing for subsequent Cre/lox recombination. Both shRNA and cDNA expression vectors also contained attL sites necessary for recombination with attR sites in our destination expression vectors. As proof of principle we demonstrate the functionality and efficiency of this system by testing expression of several cDNA and shRNA sequences in a number of cell lines.

**Conclusion:**

Our system is a valuable addition to already existing library of Gateway based vectors and can be an essential tool for many aspects of gene functional studies.

## Background

In the era of whole genome sequencing and proteomics, there has never been a greater need to develop versatile tools for gene functional studies. Such studies necessitate a series of genetic manipulations including overexpression and/or downregulation of genes of interest either in *in vivo *and/or *in vitro *settings. Downregulation of genes has been made possible by RNA interference (RNAi) technology [1] and targeting genes using expressed short hairpin RNA [2,3] is currently the method of choice by a majority of researchers. The most successful RNAi libraries based on retroviral [4] and lentiviral expression [5,6] of shRNAs have also been utilized for large-scale functional genomic screens [6].

Many gene expression platforms have been developed over years that allow for the constitutive or inducible expression of genes/shRNAs [2,3]. Furthermore, several systems for the downregulation or overexpression of multiple targets have been adapted to address issues such as isoform redundancy, endogenous mutations of single shRNAs and successful targeting of signaling pathways [7-19].

Expression platforms have been constructed either using classical restriction-enzyme based cloning technology or novel recombination based technologies, of which the Gateway technology has gained an unprecedented use. Gateway utilizes elements of site-specific recombination of *Escherichia coli *bacteriophage lambda integrase/*att *to enable the transfer of genes between different vectors [20,21]. While many varieties of Gateway-compatible vectors are available, problems may be encountered when either additional markers and/or different expression platforms are needed. This is especially the case for *in vitro *transformation experiments that are designed to introduce multiple genetic aberrations to cell lines in a stepwise fashion.

To enable efficient transfer of genes/shRNAs through different expression platforms, we have combined aspects of the Gateway system and restriction-based cloning technology and designed two entry vectors for either double shRNA or Cre/lox inducible gene expression and a series of lentiviral destination vectors containing an array of markers. We took advantage of already available and widely used expression/shRNA vectors to facilitate the conversion of reagents from one platform to the other. We have generated an additional, fully compatible system with currently available Gateway vectors which should further facilitate gene functional studies.

## Results and discussion

### Design of modified Gateway shRNA/gene expression system

**Figure 1:**
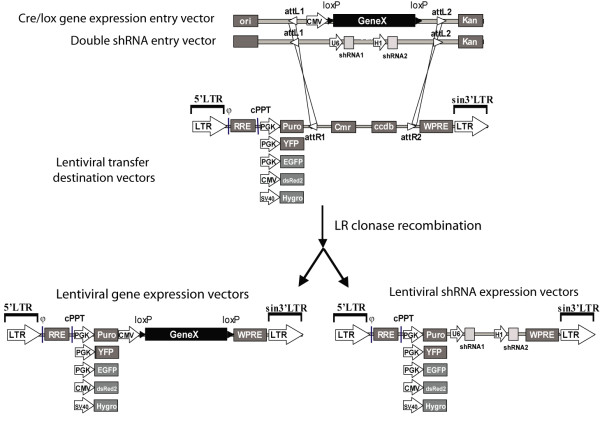
**Schematic diagram of shRNA/cDNA lentiviral expression platform**. This system consists of two entry vectors, pENTR.hU6.hH1 and pENTR.CMV.ON and six lentiviral destination vectors containing different selection markers. Entry vector insert is flanked by attL sites used for recombination with attR sites found in the lentiviral destination vectors. C/LTR, fusion between the CMV IE gene enhancer and the promoter of the HIV 5'/LTR; ψ, RNA packaging signal; *hPGK*, human phosphoglycerate kinase eukaryotic promoter; *CMV*, cytomegalovirus promoter; *SV40*, simian-virus 40 promoter, *puro*, puromycin resistance gene; *hygro*, hygromycin resistance gene;; *YFP*, yellow fluorescent protein; *EGFP*, enhanced green fluorescent protein; *dsRed2*, red fluorescent protein; *SIN/LTR*, 3' self inactivating long terminal repeat; *RRE*, Rev response element; *WPRE*, 600-bp post-transcriptional regulatory element from Woodchuck hepatitis virus.

We utilized a combination of restriction-based cloning and highly versatile Gateway site-directed recombination technology for the construction of modified gene/shRNA expression system. This system consists of two main components: gene/shRNA entry and lentiviral destination vectors (Figure 1). Shuffling of inserts from entry vectors to any of lentiviral destination vectors is mediated by LR recombination reaction. Cloning details are provided in the Materials and Methods.

The double shRNA entry vector, pENTR.hU6.hH1, was designed by combining shRNA promoter cassettes from two published shRNA expression vectors, pSUPER.retro and plko.1.puro. One advantage of this strategy is the availability of shRNA cassettes based on the subcloning into pSUPER.retro or plko.1.puro. These cassettes are used for construction of the most extensively used shRNA libraries [4,5]. While these libraries have been applied successfully over the last 5 years, one of their major limitations is the extent of available selection markers. This is often the case in cell lines that require multiple shRNA or gene expression manipulations. Therefore, subcloning of shRNA libraries in a more versatile vector that allows for downstream transfer of shRNA cassettes to various expression platforms is highly desirable. Hence, the construction of pENTR.hU6.hH1 would allow for easy transfer of subcloned shRNA cassettes to different gateway based expression vectors, shRNA targeting of multiple genes, and the increased knockdown of a single target using multiple shRNA sequences. Furthermore, since pENTR.hU6.hH1 vector contains two different shRNA expression promoters, this would possibly prevent subsequent endogenous recombinations known to occur in shRNA vectors with the same tandem promoters [16].

Gene expression vector pENTR.CMV.ON is based on Cre/lox recombination and contains CMV promoter and MCS flanked by loxP sites. Most of the entry vectors available to date are promoterless and are recombined in promoter containing expression vectors. In addition, subcloning into these vectors can be accomplished by attB-PCR recombination avoiding restriction-based cloning. While these vectors have been extensively used, they might not be adequate for certain applications. For example, attB-PCR requires very long primer sequences which sometimes results in labor intensive and time consuming PCR troubleshooting especially during amplifications of large DNA fragments. This might inadvertently increase a chance of possible transgene mutations arising during the PCR reaction. Hence, researchers would still have to resort to a more accurate restriction enzyme-based cloning. Furthermore, gene functional studies very frequently require the inducible transgene expression. To this end, we incorporated Cre/LOX technology to our gene expression platform. The final entry vector, pENTR.CMV.ON (Figure 1), contains a potent CMV promoter which provides a better chance of gene overexpression as well as loxP sites required for Cre recombinase excision. To our knowledge, this is the first entry vector available for Cre/lox based gene expression.

The destination vectors constructed in this study (Figure 1) are based on the lentiviral gene expression which is the most utilized system to efficiently transduce hard to transfect cell lines or primary mammalian cells. Our destination vectors were engineered to express an array of selection markers through transgene independent promoters. While our system was under construction, two additional versatile Gateway based expression systems were reported [19,22]. Zhu et al. [19] constructed bicistronic lentiviral gateway destination vectors with internal-ribosome entry site (IRES) which co-express the miR-shRNA with a variety of selection markers. While IRES has many advantages for the co-expression of transgenes, there have been some major limitations reported regarding their use. Several reports have shown that the IRES level of transgene expression depended considerably on the type of targeted cell [23,24]. Additionally, it was shown that IRES dependent translation could be affected by the first cistron with certain cistrons having inhibitory activity on IRES through yet undefined mechanisms [24,25]. Martin et al. [26] have also reported that several commercially available bicistronic vectors based on the IRES from Encephalomyocarditis virus (EMCV) have the 11^th ^AUG modified to a Hind*III *site to allow for easier subcloning [26]. However, this modification decreases dramatically the expression of the IRES-controlled coding sequence. Thus, our collection of lentiviral destination vectors will provide an alternative option to researchers faced with low level expression of the IRES-controlled transgene.

Campeau *et al*. have similarly developed an extensive array of lentiviral destination vectors [22] including promoterless pLentiX1 series which are compatible with our entry vectors. However, in this work we have constructed additional destination vectors expressing YFP, hygro and dsRed2 markers.

### Functionality of shRNA double promoter expression entry vector

**Figure 2:**
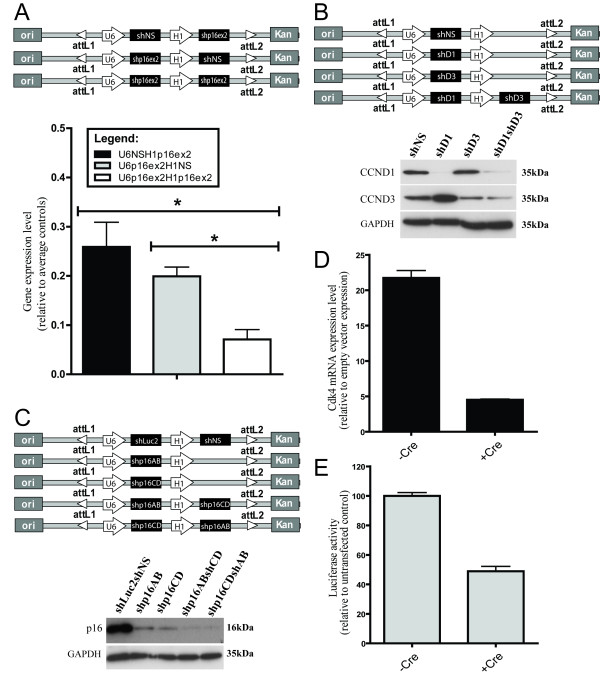
**Successful shRNA/cDNA targeting using designed entry vectors**. A) pENTR constructs targeting exon 2 of p16 were transfected into 293T cells. RNA was isolated 48 hours post transfection and the mRNA expression level was determined using Q-RT-PCR. The average represents fold gene expression level relative to pENTRhU6hH1 transfected 293T cells (Students t-test; p < 0.01). B) pENTR constructs for CCND1 and/or CCND3 shRNA expression were recombined with plko.puro.DEST. Western blot shows the successful knockdown of CCND1/CCND3 in Panc1 cells. C) pENTR constructs for p16 shRNA expression were recombined with plko.YFP.DEST. Western blot shows p16 knockdown in H6c7 cells transfected with p16 shRNA constructs. D) pENTR.Cre mediated Cdk4 overexpression in 293T cells transiently transfected with pENTR.Cdk4.ON. The average represents fold gene expression level relative to control vector cell line f) Luciferase activity downregulation by Cre protein in stable HeLa cells overexpressing Luc2 mediated by plko.dsRed2.Luc2.ON lentivirus.

To demonstrate the efficacy of pENTR.hU6.hH1 construct we assessed its silencing effect on single and multiple gene targets. To test the functionality of both hU6 and hH1 promoters, we created three double promoter shRNA expression vectors with either shRNA targeting exon 2 of p16^INK4a ^(p16ex2) or nonsilencing shRNA (NS) under hU6 and/or hH1 (Figure 2A). These vectors were transiently transfected into 293T cells and the p16 mRNA expression levels were determined using reverse transcription-quantitative polymerase chain reaction (Q-RT-PCR). The mRNA knockdown was achieved when p16ex2 shRNA was expressed either under the hH1 or hU6 promoter when compared to control vector. Furthermore, there was a decrease in shRNA knockdown when using hH1 promoter, which is consistent with the previous reports of hH1 being a weaker promoter as compared to hU6 promoter [27]. Double expression of p16ex2 significantly decreased the p16 mRNA levels in 293T cells as compared to a single expression of p16ex2 under either promoter. The additive/double suppression of p16 mRNA could not be attributed to nonspecific effects of shRNA silencing since vectors co-expressing both p16ex2 and control NS showed lower level of p16 knockdown as compared to vector doubly expressing p16ex2.

Our next experiment to test the functionality of pENTRhU6hH1 involved creating stable cell lines that would allow enough time for any possible shRNA recombinations known to arise in the targeted cells as a result of shRNA expression [16]. If such events occur using our system, then the knockdown efficiency would be greatly impaired. To test this, we chose shRNA against single targets, cyclin D1 (CCND1) and cyclin D3 (CCND3), which has been previously published [28]. We created three vectors pENTR.shD1, pENTR.shD3, pENTR.shD1.shD3 and control vector expressing non-silencing shRNA, pENTR.shNS (Figure 2A). The resulting vectors were recombined with our destination vector pLKO.puro.DEST and lentiviruses were generated for transduction into the Panc-1 cell line. Western analysis on protein lysates from puromycin-selected Panc1 cells revealed specific knockdown of CCND1 and CCND3 with their respective shRNAs (Figure 2B). In addition, the Panc1 cell line transduced with shD1_D3 lentivirus showed significant knockdown of both cyclin D1 and cyclin D3 proteins with no significant loss of single shRNA effectiveness (Figure 2B). An upregulation of CCND3 in shD1 expressing Panc-1 cells was noted. We observed the same effects previously [28] and attributed it to a compensatory mechanism. These effects seem to be alleviated with the co-suppression of CCND3 as demonstrated in our current results (Figure 2B).

To determine whether our dual shRNA expression vector can be used to maximize the efficiency of gene silencing we targeted two different regions in exon 1 of *p16*^INK4b ^mRNA. The experiments were performed on human pancreatic duct epithelial cell line H6c7 that expresses very high levels of p16*^INK4a ^*due to loss of Rb secondary to HPV E6/E7 expression [29]. The efficient knockdown of *p16*^INK4a ^in H6c7 cells by shRNA has presented a major challenge. While we tested 16 different shRNAs against *p16*^INK4a ^(Additional file 1), none of the resulting H6c7 stable cell lines has shown greater than 60% reduction in p16*^INK4a ^*levels. Additional technical difficulty in the specific silencing of *p16*^INK4a ^is that one can only target exon 1, since exons 2 and 3 are shared with *p14*^ARF^. This limits significantly the available mRNA sequence for siRNA design. To test if our dual shRNA expression vector would overcome these problems, four entry vectors expressing either single or combination of the most effective shRNAs against *p16*^INK4a^, shp16AB and shp16CD [30] were recombined with the plko.YFP.DEST vector (Figure 2C). H6c7 cells were transduced with the resulting lentiviruses and the p16 protein levels were assessed by western blotting. As predicted, the expression of two shRNAs against *p16*^INK4a ^led to a more effective suppression of the p16*^INK4a ^*level than a single shRNA cassette (Figure 2C). We also tested the actual p16AB and p16CD shRNA expression under different promoters using Q-RT-PCR (Additional File 2). While both promoters efficiently expressed p16AB and p16CD shRNA, there was a lower siRNA expression under hH1 promoter as compared to hU6 promoter (Additional File 2). A number of factors should be considered prior to usage of the hH1 and hU6 promoters, the first being the potency of the shRNA used and the second being the cell type. The hH1 promoter should be employed for intermediate knockdown, while the hU6 can be used for more potent applications. Hence, our double shRNA expression vector can be particularly valuable for dose-dependent studies where variable expression is required.

### Functionality of Cre/lox inducible entry vector

Next, we tested the functionality and efficiency of pENTR.CMV.ON vector using both transient and stable transfections. For transient expression testing, cyclin-dependent kinase 4 (Cdk4) was subcloned into pENTR.CMV.ON vector. The resulting pENTR.Cdk4.ON and empty control vector were transfected into 293T cells. As measured by Q-RT-PCR, Cdk4 was overexpressed (21.73 ± 1.05 fold) compared to the empty vector control (Figure 2E). Additionally, co-transfection with the Cre expressing vector, pENTR.Cre, decreased the Cdk4 mRNA levels to 4.52 ± 0.11 fold relative to the control vector (Figure 2E).

To create stable clones, we constructed vector expressing luciferase protein (pENTR.Luc2.ON) and recombined it with the plko.dsRed2.DEST vector, thus creating the destination clone plko.dsRed2.Luc2.ON. Lentiviruses generated using plko.dsRed2.Luc2.ON and control plko.dsRed2.DEST vectors were used to transduce HeLa cells, which were subsequently sorted for Red2 fluorescence. Luciferase protein was successfully overexpressed in HeLa-dsRed2.Luc2.ON cells as measured by Q-RT-PCR (data not shown) and luciferase assay (Figure 2F). Transient transfection of pENTR.Cre into these cells resulted in a significantly decreased luciferase activity (Figure 2F). Taken together these results demonstrated the functionality of both pENTR.CMV.ON and plko.dsRed2.DEST vectors.

### Functionality of lentiviral destination vectors

While we successfully demonstrated the efficiency of destination vectors plko.puro.DEST, plko.YFP.DEST and plko.dsRed2.DEST (Figure 2), destination vectors expressing hygromycin (hygro) and green fluorescent protein (EGFP) remained to be tested.

**Figure 3:**
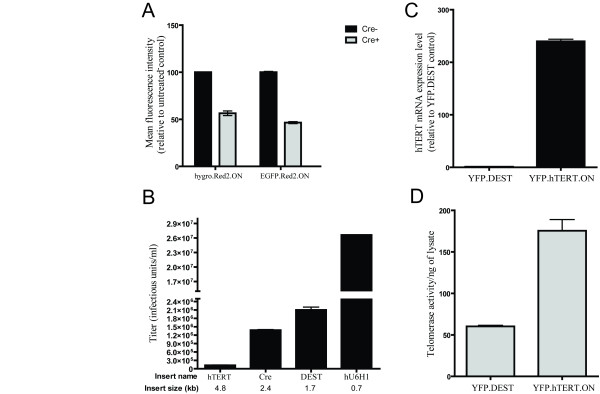
**Functionality and efficacy of lentiviral destination vectors**. A) H6c7 cells were transduced with viruses generated using dsRed2 destination expression vectors carrying EGFP or hygro selection markers. Following selection/sorting cells were infected with Adeno-Cre and subjected to flow cytometry 72 hours post infection. Red fluorescent profiles are compared between infected and non-infected cells. B) Virus titers of lentiviruses expressing increasing insert size as measured on 293T cells C) hTERT is overexpressed and D) active in H6c7 cell lines stably transduced with plko.YFP.hTERT.ON lentivirus.

To test the performance of remaining destination vectors, H6c7 stable cell lines expressing dsRed2 were generated using lentiviruses from plko.hygro.dsRed2 and plko.EGFP.dsRed2 vectors. H6c7 cells were selected or sorted for their respective selectable markers and changes in red fluorescence were measured by flow cytometry. The plko.hygro.dsRed2 construct conferred resistance to hygromycin while plko.EGFP.dsRed2 expressed EGFP (data not shown). Furthermore, all H6c7 dsRed2 derived cell lines were positive for red fluorescence suggesting that dsRed2 could be successfully overexpressed using any of our destination vectors (Figure 3A). The infection of H6c7 dsRed2 derivatives using Adeno-Cre significantly reduced the expression of dsRed2 as measured by Red2 fluorescence (Figure 3A).

### Viral titer of destination vectors is dependent on the insert size

It has been demonstrated that lentiviral titer depends greatly on the insert sequence length due to the packaging capacity of the lentivirus [31,32]. As a result multiple strategies have been employed over years to increase the viral titers of pseudotyped lentiviruses especially for gene therapy applications [33-36]. In order to fully characterize the efficacy of our expression system, we tested the viral titer of plko.YFP.DEST expressing genes of various sizes in 293T cells (Figure 3B). As predicted, our results showed that the viral titer is proportional to the insert size (Figure 3B). Although a destination vector with a large cDNA such as hTERT resulted ultimately in lower lentiviral titer, it did not impede the ability to efficiently overexpress the gene in stable H6c7 cell line (Figure 3C). Furthermore, it has been shown previously that low titer virus results in unprocessed and defective lentiviruses [35]. However, in our experiments, all YFP positive cells clearly overexpressed functional hTERT gene as determined by the hTERT activity assay (Figure 3D). Since the chance of vector integration event using non-viral expression system is generally very low, the use of low titer lentiviruses might still remain a better choice when creating stable cell lines.

## Conclusion

Versatile gene/shRNA expression system is a necessity in gene functional studies. We have created a modified gateway lentiviral system for shRNA and Cre/lox inducible gene expression with entry and destination vectors that are compatible with existing gateway technologies. These reagents should provide additional choices for researchers studying many aspects of functional genomics.

## Methods

### Vector construction

All detailed maps and sequences of vectors constructed in this study are available upon request. Construction strategies, vector maps and restriction enzyme analysis were performed using Vector^NTI ^software (Invitrogen, Carlsbad, CA).

### Entry vectors construction

The pENTR.hU6.hH1 was constructed in two steps. In the first step, *Xho*I digested hU6 cassette from plko.1-puro [37] was ligated into *Xho*I/*Sal*I linearized pENTR-4 vector (Invitrogen, Carlsbad, CA) to construct an intermediate single shRNA expression vector pENTR.hU6. Then, *EcoR*I/*Xho*I digested hH1 cassette from pSUPER-retro [38] was inserted into *EcoR*I/*Xho*I linearized pENTR.hU6 vector creating double shRNA expression vector pENTR.hU6.hH1. We also constructed a second single shRNA expression vector pENTR-hH1 by removing hU6 cassette from pENTR.hU6.hH1 with *Not*I/*Eco*RI. Blunt ends were then generated by Klenow reaction and subsequently ligated to make pENTR.hH1.

The pENTR.CMV.ON was constructed by inserting *EcoR*V/*Sna*BI linker cassette from pSicoR-puro-linker-loxp plasmid containing MCS flanked by loxP sites into *Hind*III/*Sna*BI linearized pENTR.CMV vector. *Hind*III site was blunt ended using Klenow reaction. Both intermediate plasmids pSicoR-puro-linker-loxp and pENTR.CMV vectors were constructed in our laboratory (data not shown).

### Destination vectors

The plko.puro.DEST was constructed by inserting *EcoR*V/*Mlu*I digested attR1-cmr-ccdb-attR2 cassette from pTREX-DEST30 (Invitrogen) into *Cla*I/*Mlu*I digested plko.1-puro vector (*Cla*I blunt ended with Klenow enzyme). The plko.YFP.DEST was constructed by linearizing plko.YFP (gift from Dr. Linda Penn, Ontario Cancer Institute, Canada) with *Age*I/*EcoR*I and ligating *Age*I/*Nhe*I digestedattR1-cmr-ccdb-attR2 cassette from pTREX-DEST30. The *EcoR*I and *Age*I sites were blunt ended using Klenow enzyme. The plko.dsRed2.DEST was made by inserting *Ssp*I/*Nsi*I dsRed2 cassette from pSUPER.dsRed2 (constructed in our laboratory) into the *Xba*I (blunt ended)/*N*siI digested plko.puro.DEST vector. The Destination vector plko.hygro.DEST was constructed in two steps. In the first step, an intermediate plasmid pBS-hygro was created by subcloning *Xmn*I/*Sal*I hygro cassette from *Sal*I/*Eco*RV pIND/Hygro (Invitrogen) into pBluescriptIISK+ (Stratagene). Next, *Kpn*I/*Spe*I hygro cassette from pBS-hygro was subcloned into plko.puro.DEST linearized with *Kpn*I/*Spe*I creating plko.hygro.DEST vector. To construct plko.EGFP.DEST, the EGFP PCR fragment was (EGFP: F:5'TAGAATTCTACCGGGTAGGGGA3'; R: GGGGGTACCTCATTGGTCTTAAAGGTACCGA5') digested with the *KpnI *restriction enzyme at the 3' end and were then subcloned into the *Xcm*I/*Kpn*I linearized plko.puro.DEST plasmid.

### Subcloning into the entry vectors

The pENTR.Luc.ON was constructed by inserting *Nhe*I/*Xho*I luciferase fragment from pIND/HygroLuc (Clontech, Mountain View, CA) into *Avr*II/*Xho*I linearized pENTR-CMV-ON. The pENTR-dsRed2-ON was obtained by ligating dsRed2 fragment (*Hinc*II/*Xho*I) into *Hpa*I/*Xho*I linearized pENTR-CMV-ON.

Oligos coding for the various shRNAs were annealed and cloned into either *Bgl*II/*Hind*III or *Age*I/*EcoR*I- digested pENTRhU6hH1 as described previously [2]. All shRNAs used in this study are listed in Additional file 1.

### Transfer of cDNA/shRNA cassettes to lentiviral destination vectors

The constructs were designed to permit subcloning of shRNA/cDNA cassettes to different expression platforms by Gateway recombination. This involved direct LR recombination (Invitrogen, San Diego, CA) of the promoter containing shRNA/cDNA entry vector to any one of promortless lentiviral destination vectors with expression of different markers or drug selection genes.

### Bacterial strains and transformation

For the construction of entry and destination vectors containing a toxic *ccdB *gene competent *Escherichia coli *Library Efficiency DB3.1™cells (Invitrogen), were used for propagation according to the manufacturer's recommendations. For construction of other vectors and clones not containing the *ccdB *gene, SUBCLONING EFFICIENCY™DH5α competent cells (Invitrogen) were used for transformation according to the supplier's instruction

### Cell culture

HeLa, Panc1 and 293T cells were obtained and cultured as recommended by the American Type Culture Collection (Rockville, MD). The Human pancreatic duct epithelial cell line (H6c7) was cultured as described previously [29].

### Transient transfections and stable cell line generations

For transient transfection assays cells were transfected using Lipofectamine reagent (Invitrogen, Carlsbad, CA). For virus preparations, 293T cells were transfected using BD CalPhos Mammalian Transfection Kit (BD Biosciences). Both assays were performed as per the protocol provided by the manufacturer.

### Virus preparation, transduction and stable cell line generation

Lentiviruses were prepared by transfecting three plasmids into 293T cells as described previously [39]. The plasmids are pMDLg/pRRE, the vesicular stomatitis virus (VSV-G) envelope plasmid pCMV-VSG, rev expressing plasmid pRSV-Rev and destination vectors containing the self-inactivating LTR. Stocks were stored frozen at -80°C and tittered on 293T cells.

Stable cell lines were isolated following viral transductions by either selection in the appropriate antibiotics for 1-2 weeks (0.5 μg/mL puromycin; 50 μg/mL hygromycin; 100 μg/mL) or by flow sorting using dual-laser FACSCalibur.

Adenovirus expressing Cre recombinant protein was purchased from Vector Biolabs (Philadelphia, PA) and used at 10 moi per cell for 72 hours.

### RNA isolation and Q-RT-PCR analysis

RNA isolation and assay techniques used in this paper were published in our previous work [28]. Primers used in this study are listed in Additional file 1.

### Western analysis

Immunoblotting was performed using whole protein extracts and probed with the following antibodies against cyclin D3 (BD Biosciences, San Jose, CA), cyclin D1 (BD Biosciences), p16^INK4a ^(Cell Signalling, Boston, MA) and p14^ARF ^(NeoMarkers, Freemont, CA), GAPDH (Abcam, Cambridge, MA), and secondary mouse or rabbit conjugated IgG-horseradish peroxidase (Cell Signalling).

### Luciferase and hTERT assay

Luciferase assay was performed using Luciferase Assay Kit (Promega Madison, WA). The hTERT activity was measured using TRAPEZE^® ^RT Telomerase Detection Kit (Millipore, Billerica, MA). Both kits were used as recommended by the manufacters protocols.

### Flow cytometry

The fluorescent emission intensities of EGFP, YFP and dsRed2 proteins were measured by FACS analysis using the FACSCalibur™(BectonDickinson Corp.). Data were acquired and analyzed using CellQuest™v.3.0.

## Competing interests

The authors declare that they have no competing interests.

## Authors' contributions

NR designed and constructed majority of the vectors, carried out the proof-of-principle studies, and drafted the manuscript. LL constructed plko.EGFP.DEST and pENTR.Red2.ON vectors and assisted in drafting the manuscript. MT conceived and provided overall supervision of the study and edited the manuscript. All the authors have read and approved the final manuscript.

## Supplementary Material

Additional file 1 **shRNA sequences and Q-RT-PCR primers used in this study**. Click here for file

Additional file 2 **Figure S1: Comparison of shRNA expression under**. Click here for file
