# Supplementary material for: Modified gateway system for double shRNA expression and Cre/lox based gene expression
Source: BMC Biotechnol. 2011 Mar 22;11:24. doi: 10.1186/1472-6750-11-24 (PMC3070635; doi:10.1186/1472-6750-11-24)
Supplement: Additional file 1 — shRNA sequences and Q-RT-PCR primers used in this study. [file 1472-6750-11-24-S1.DOCX]

Additional file 1

Supplementary Table 1: shRNA sequences used in this study

| **shRNA name** | **Target gene** | **Target sequence** |
| --- | --- | --- |
| p16nt_37 | CDKN2A | AAGTGCTCGGAGTTAATAGCA |
| p16nt_385 | CDKN2A | AACGCACCGAATAGTTACGGT |
| p16nt_741 | CDKN2A | AAAGAACCAGAGAGGCTCTGA |
| p16nt_235 | CDKN2A | GCATGGAGCCTTCGGCTGACT |
| p16nt_400 | CDKN2A | GGCGACTCTGGAGGACGAAGT |
| p16nt_540 | CDKN2A | TGGGAAACCAAGGAAGAGGAA |
| p16nt_1252 | CDKN2A | GCGCACATTCATGTGGGCATTT |
| p16nt_288 | CDKN2A | GAGGAGGTGCGGGCGCTGC |
| p16nt_336 | CDKN2A | AATAGTTACGGTCGGGAGGCC |
| p16nt_957 | CDKN2A | AAAGAACCAGAGAGGCTCTGA |
| p16ex2 | CDKN2A | AAACTTAGATCATCAGTCACCGA |
| p16nt_1138 | CDKN2A | AAATGTCCATTTATATCATTTTT |
| p16nt_1118 | CDKN2A | AAACACCGCTTCTGCCTTTTCAC |
| p16nt_329 | CDKN2A | CGCACCGAATAGTTACGGT |
| p16AB | CDKN2A | GGGAGCAGCATGGAGCCTTCGG |
| p16CD | CDKN2A | GGTCGGGTAGAGGAGGTGCGGG |
| D1_1 | Cyclin D1 | GTTCGTGGCCTCTAAGATGAA |
| D3_1 | Cyclin D3 | ACAGAATTGGATACATACACC |
| Luc2 | Luciferase | GTGCCAGAGTCCTTCGATTCC |
| NS | Non specific | ATTCTATCACTAGCGTGAC |

Supplementary Table 2: Primers for Q-RT-PCR

| **Primer** | **Forward** | **Reverse** |
| --- | --- | --- |
| CCND1 | AGGTCTGCGAGGGAACAGAAGTG | TGCAGGCGGCTCTTTTTC |
| CCND3 | GCAGCGCCTTTCCCAACT | TCAAAAGGAATGCTGGTGTATGTATC |
| CRE | ACGACCAAGTGACAGCAATG | CACCGTCAGTACGTGAGATA |
| hTERT | TTCAAGGCTGGGAGGAACAT | TGACACTTCAGCCGCAAGAC |
| Cdk4 | ACATGTGGAGTGTTGGCTGTATCT | GCAGCCCAATCAGGTCAAA |
| p16 exon 1 | CCAACGCACCGAATAGTTACG | CCATCATCATGACCTGGATCG |
| RPS13 | GTTGCTGTTCGAAAGCATCTTG | AATATCGAGCCAAACGGTGAA |
