# Supplementary material for: Modified gateway system for double shRNA expression and Cre/lox based gene expression
Source: BMC Biotechnol. 2011 Mar 22;11:24. doi: 10.1186/1472-6750-11-24 (PMC3070635; doi:10.1186/1472-6750-11-24)
Supplement: Additional file 2 — Figure S1: Comparison of shRNA expression under. [file 1472-6750-11-24-S2.PDF]

A

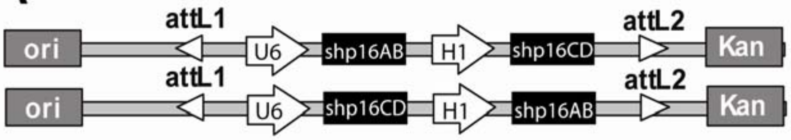

B

	shp16AB	shp16CD
hU6	24.24±0.0153	22.97±0.060
hH1	24.53±0.0666	23.48±0.065

C

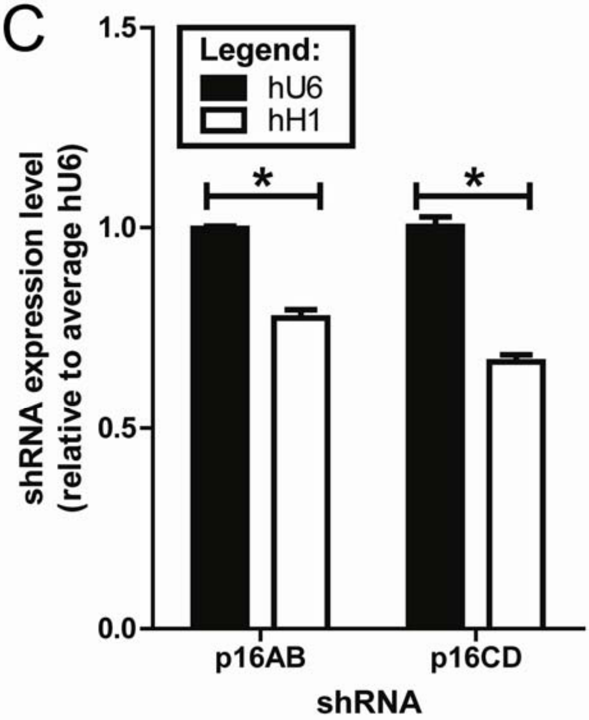

Figure S1. Comparison of siRNA levels expressed under different promoters. A) Double expression pENTR constructs expressing p16AB and p16CD shRNA under hU6 or hH1 promoter were transfected into 293T cells. B) Total RNA was isolated 48 hours post transfection and the poly-A tail was added to the 3' end of small non-coding RNAs using QuanitMiR RT Kit (System Biosciences, CA, USA). Following reverse transcription with oligo-dT primer, the resulting cDNA was subjected to Q-RT-PCR analysis using siRNA specific forward primer and universal reverse primer. B) Average ct±stdev for each shRNA are depicted in the table. To control for RNA input, the expression levels were normalized to endogenous expression levels of the human miR-16. C) The siRNA expression levels are depicted as ΔΔCt levels relative to average ΔCt siRNA levels expressed under hU6 promoter (Two-way ANOVA; $p<0.01$).
